# Prophylactic efficacy against *Mycobacterium tuberculosis* using ID93 and lipid-based adjuvant formulations in the mouse model

**DOI:** 10.1371/journal.pone.0247990

**Published:** 2021-03-11

**Authors:** Susan L. Baldwin, Valerie A. Reese, Sasha E. Larsen, Elyse Beebe, Jeff Guderian, Mark T. Orr, Christopher B. Fox, Steven G. Reed, Rhea N. Coler

**Affiliations:** 1 Seattle Children’s Research Institute, Seattle, WA, United States of America; 2 Infectious Disease Research Institute, Seattle, WA, United States of America; 3 Department of Global Health, University of Washington, Seattle, WA, United States of America; Colorado State University, UNITED STATES

## Abstract

An estimated 10 million people developed tuberculosis (TB) disease in 2019 which underscores the need for a vaccine that prevents disease and reduces transmission. The aim of our current studies is to characterize and test a prophylactic tuberculosis vaccine comprised of ID93, a polyprotein fusion antigen, and a liposomal formulation [including a synthetic TLR4 agonist (glucopyranosyl lipid adjuvant, GLA) and QS-21] in a preclinical mouse model of TB disease. Comparisons of the ID93+GLA-LSQ vaccines are also made to the highly characterized ID93+GLA-SE oil-in-water emulsion adjuvant, which are also included these studies. The recent success of vaccine candidate M72 combined with adjuvant AS01_E_ (GlaxoSmithKline Biologicals) in reducing progression to active disease is promising and has renewed excitement for experimental vaccines currently in the TB vaccine pipeline. The AS01_E_ adjuvant contains monophosphoryl lipid A (MPL) and QS-21 (a saponin) in a liposomal formulation. While AS01_E_ has demonstrated potent adjuvant activity as a component of both approved and experimental vaccines, developing alternatives to this adjuvant system will become important to fill the high demand envisioned for future vaccine needs. Furthermore, replacement sources of potent adjuvants will help to supply the demand of a TB vaccine [almost one-quarter of the world’s population are estimated to have latent *Mycobacterium tuberculosis* (Mtb) according to the WHO 2019 global TB report], addressing (a) cost of goods, (b) supply of goods, and (c) improved efficacy of subunit vaccines against Mtb. We show that both ID93+GLA-SE (containing an emulsion adjuvant) and ID93+GLA-LSQ (containing a liposomal adjuvant) induce ID93-specific TH1 cellular immunity including CD4+CD44+ T cells expressing IFNγ, TNF, and IL-2 (using flow cytometry and intracellular cytokine staining) and vaccine-specific IgG2 antibody responses (using an ELISA). In addition, both ID93+GLA-SE and ID93+GLA-LSQ effectively decrease the bacterial load within the lungs of mice infected with Mtb. Formulations based on this liposomal adjuvant formulation may provide an alternative to AS01 adjuvant systems.

## Introduction

The only vaccine currently available for use against Mtb is the attenuated live vaccine, bacille Calmette-Guérin (BCG), which is known to reduce childhood TB (disseminated extrapulmonary Mtb) but wanes with time, giving variable efficacy against adult TB (pulmonary Mtb) [1]. Efforts are currently underway for the development of safer alternatives to the BCG vaccine due to the potential of the attenuated live BCG vaccine to cause disseminated BCG disease in immunocompromised people, such as those with HIV [2, 3]. The lack of a surrogate immune signature that defines vaccine efficacy against Mtb infection or TB disease has, however, made it challenging to accelerate promising vaccine candidates. Even so, the recent clinical prevention of disease (POD) trial for TB with the M72 subunit vaccine candidate adjuvanted with a liposomal formulation including monophosphoryl lipid A (MPL) and QS-21 (M72/AS01_E_, GlaxoSmithKline Vaccines) has shown promise [4]. The phase 2b clinical results of the M72/AS01_E_ vaccine showed 54% protective efficacy against active pulmonary TB disease [4] and remains nearly 50% effective (49.7%) for at least 3-years after the final boost immunization [5]. These results have generated immense enthusiasm for the feasibility of additional subunit vaccines and adjuvants that could also prove to be effective.

The design and development of successful subunit TB vaccine candidates will require antigens and adjuvants that are immunogenic, inexpensive, and accessible to remote regions of the world [6, 7]. The well-characterized synthetic TLR4 agonist glucopyranosyl lipid adjuvant (GLA) [8] has successfully been used in several clinical trials (nearly 3,000 individuals to date) for use in vaccines targeted against a variety of infectious diseases [9–13]. Herein, the aim of this work was to characterize the prophylactic capacity of ID93 combined with GLA formulated in a QS-21-containing liposomal formulation (GLA-LSQ) in the tuberculosis mouse model. The fusion antigen ID93 is comprised of four Mtb proteins covering a breadth of disease stages of an infection: Rv2608 (PE/PPE family), Rv1813 (expressed under stress/hypoxia), Rv3619 (esxV, an ESAT-6 like protein 1), and Rv3620 (esxW; ESAT-6 like protein 10) [14], whereas M72 includes only two Mtb proteins [Mtb39A (Rv1196, PPE18) and Mtb32A (Rv0125, serine proteinase)] [15].

Demand for adjuvants such as AS01 (GSK) may expand since it is a component of the highly successful FDA approved Shingrix vaccine (affording over 90% efficacy) and the RTS,S/AS01 Mosquirix malaria vaccine [approved by the European Medicines Agency (EMA)] [16]. The mechanism of action of the QS-21 component of AS01 and the development of semi-synthetic analogs of the natural QS-21 product are reviewed in detail elsewhere [16]. The main structural and biological differences between monophosphoryl lipid A adjuvant (MPL), which is included in the AS01_E_ adjuvant, and GLA have been published [8]. Importantly, whereas MPL is a heterogeneous mixture derived from a biological source, *Salmonella minnesota* R595, GLA is a pure, synthetic hexaacylated lipid A [8]. A description of the liposomal formulation of AS01_E_ has also been published, and comprises liposomes with MPL, and QS-21 bound to cholesterol, which reduces its hemolytic activity [17].

Here we expand on our previous preclinical work by investigating the use of vaccine candidate ID93 with liposomal formulations of GLA (glucopyranosyl lipid adjuvant) to determine the prophylactic efficacy potential. As with the pre-clinical data collected for ID93+GLA-SE (GLA in a stable oil-in-water emulsion), the work done in these studies with ID93+GLA-LSQ will help with future clinical trial design for a POD vaccine against Mtb. An effective, readily available POD vaccine could dramatically reduce the morbidity and mortality associated with Mtb infections worldwide.

## Materials and methods

### Antigen and adjuvants

The ID93 recombinant fusion protein is comprised of the Mtb proteins Rv2608, Rv3620, Rv1813 and Rv3619 and was prepared at IDRI (Seattle, WA) as previously described [14]. The stock concentration of ID93 was 0.69 mg/ml and was diluted immediately before immunization with saline.

GLA was obtained from Avanti Polar Lipids. QS-21 was prepared at IDRI (Seattle, WA) from semi-purified saponin (Quil A from Brenntag Biosector, Frederikssund, Denmark) or obtained directly from Desert King International (San Diego, CA). Briefly, liposomes were manufactured by combining lipid components and GLA in organic solvent consisting of chloroform or chloroform:methanol, which was then evaporated on a rotary evaporator or centrifugal rotary evaporator overnight. The dried components were rehydrated in the indicated buffer and placed in a sonicating water bath at ~60°C until all the solids were suspended in the buffer. The mixture was then transferred to a Microfluidics M110P (Newton, MA) for high-pressure homogenization for 5–12 passes at 10–30 kpsi. All formulations were stored at 5°C following manufacture. Formulations were characterized by dynamic light scattering (particle diameter) and HPLC (adjuvant content) (Table 1) as previously described [18, 19]. GLA-AF, GLA-SE, and SE formulations were prepared as previously described [18]. Antigen was admixed with adjuvant formulation immediately prior to immunization. Thus, antigens were not encapsulated in the liposomes. Although antigen-adjuvant association was not assessed as part of the current report, our previous work [20] indicates that the ID93 antigen tends to associate with GLA-SE but not GLA-AF or anionic GLA liposomes (equivalent to GLA-LS2 in the current report).

**Table 1:** Adjuvant nomenclature and characteristics.

Vaccine	Antigen (ID93) Dose	Adjuvant Dose	Adjuvant Description	Particle Diameter of Representative Adjuvant Batch (Z-ave, nm)
ID93+SE	-		Stable oil-in-water emulsion	82
ID93+GLA-SE	0.5 μg	5 μg GLA	GLA + stable oil-in-water emulsion	77
ID93+GLA-AF	0.5 μg	5 μg GLA	GLA aqueous nanosuspension	41
ID93+LS	0.5 μg		Neutral liposomes	87
ID93+LSQ	0.5 μg	2 μg QS-21	Neutral liposomes + QS-21	87
ID93+GLA-LS1	0.5 μg	5 μg GLA	GLA + PEGylated liposomes	58
ID93+GLA-LSQ1	0.5 μg	5 μg GLA	GLA + PEGylated liposomes + QS-21	59
		2 μg QS-21		
ID93+GLA-LS2	0.5 μg	5 μg GLA	GLA + Anionic liposomes	73
ID93+GLA-LSQ2	0.5 μg	5 μg GLA	GLA + Anionic liposomes + QS-21	70
		2 μg QS-21		
ID93+GLA-LS3	0.5 μg	5 μg GLA	GLA + Neutral liposomes	77
ID93+GLA-LSQ3 (GLA-LSQ)	0.5 μg	5 μg GLA, 0.4, 2, or 10 μg QS-21	GLA + Neutral liposomes + QS-21	75

DPPC (1,2-dipalmitoryl-sn-glycero-3-phosphocholine) and DPPG (1,2-dipalmitoryl-sn-glycero-3phospho(1’-rac-glycerol)) were obtained from Lipoid LLC, Newark, NJ. DSPE-PEG2000 (1,2-distearoyl-sn-glycero-3-phosphoethanolamine-N-[methoxy(polyethylene glycol)-2000] was purchased from CordenPharma International. Plant-derived cholesterol was acquired from Sigma-Aldrich Fine Chemicals (SAFC), St. Louis, MO, and buffer salts were obtained from J.T. Baker (Phillipsburg, NJ). Following previously described procedures[18], GLA was formulated in an aqueous nanosuspension (GLA-AF; aqueous formulation), stable emulsion (GLA-SE), or in one of three different liposome compositions. The three liposome compositions were prepared as follows: PEGylated liposomes (GLA-LS1 [3.3:1:1 weight ratio of DPPC:DSPE-PEG2000:cholesterol in 25 mM ammonium phosphate buffer]), anionic liposomes (GLA-LS2 [(3.3:0.4:1 weight ratio of DPPC:DPPG:cholesterol in 25 mM ammonium phosphate buffer]), or DOPC 1,2-dioleoyl-sn-glycero-3-phosphocholine neutral liposomes (GLA-LS3 [4:1 weight ratio of DOPC:cholesterol in 25 mM ammonium phosphate buffer]). In addition, each liposome composition was optionally formulated with QS-21 by adding aqueous QS-21 to the prepared liposome (e.g. GLA-LSQ1 for PEGylated QS-21 liposomes, GLA-LSQ2 for anionic QS-21 liposomes, GLA-LSQ3 for neutral QS-21 liposomes). In general, liposomes were prepared as 2x or 4x concentrate and mixed with antigen and diluent prior to administration. A description of the vaccines included within this manuscript is included in Table 1.

### Human subjects

All human blood research reported here was reviewed and approved by Western Institutional Review Board. IRB approval was in place prior to grant funding (reference Western Institutional Review Board File #20020527). All human subjects undergo an IRB-approved informed consent process involving; (i) provision to subjects with adequate information to allow for an informed decision about participation in the clinical investigation; (ii) facilitating the potential subject’s comprehension of the information; (iii) providing adequate opportunity for the potential subject to ask questions and to consider whether to participate; (iv) obtaining the potential subject’s voluntary written agreement to participate; and (v) continuing to provide information as the clinical investigation progresses or as the subject or situation requires. For the in vitro studies, human blood samples were collected from normal, healthy adult donors using standard phlebotomy techniques. Human peripheral blood collected by standard venipuncture was obtained from study subjects recruited in Seattle by IDRI.

#### Human subjects involvement and characteristics

Human blood samples are necessary for the proposed studies. The study population included adults of both genders. Most study volunteers in the Seattle area are Caucasians (of European origin different than Spanish), African Americans, and Asians. The area of study does not have a significant population of American Indian or Hispanics.

#### Sources of materials

The blood materials were obtained from adult volunteers in Seattle through IDRI’s Research Donor Program. These volunteers are recruited by word-of-mouth from IDRI, University of Washington and local health care institutions.

Written consent was obtained with the research subject receiving a copy of the signed and dated informed consent.

### Human whole blood *in vitro* stimulation

Adjuvant formulations were diluted in irrigation-grade saline and added to 96-well U-bottom tissue culture plates. Heparinized whole blood, obtained from healthy donors, was added in duplicate to each formulation, resulting in final GLA concentrations of 10μg/mL or 1μg/mL. Plasma supernatants were harvested after 24 hours of culture at 37°C then assayed for Tumor necrosis factor-alpha (TNFα), Interleukin-6 (IL-6), Interleukin-1-beta (IL-1β), Interferon-gamma (IFN-γ), C-C motif chemokine ligand 2 (CCL2), C-X-C motif chemokine ligand 8 (CXCL8) (eBioscience, San Diego, CA), C-C motif chemokine ligand 4 (CCL4), C-X-C motif chemokine ligand 5 (CXCL5) and C-X-C motif chemokine ligand 10 (CXCL10) (R&D Systems, Minneapolis, MN). Duplicate wells for each donor and condition were averaged for a single value.

### Human dendritic cell generation and stimulation

Whole blood was obtained from healthy volunteers and peripheral blood mononuclear cells (PBMC) were isolated by standard Ficoll-Hypaque density gradient centrifugation. CD14+ monocytes were magnetically labeled with CD14 MicroBeads (Miltenyi Biotec, Auburn, CA) and purified on a magnetic-activated cell sorting (MACS) separator following the manufacturer’s protocol. The eluted fraction containing the CD14+ cells were cultured at 5–6 x 10^6^ cells per well in 6-well tissue culture plates using complete medium (RPMI 1640, 10% fetal bovine serum, 1% L-glutamine, 1% penicillin/streptomycin) supplemented with GM-CSF (50 ng/mL) and IL-4 (50 ng/mL). After incubating for 5 days at 37°C, the cells were washed, counted, and plated in 96-well U-bottom tissue culture plates at 6 x 10^4^ cells per well, and incubated with formulations diluted to 10 μg/mL and 1 μg/mL of GLA, in duplicate. After incubating at 37°C for 24 hours, supernatants were assayed for TNFα, IL-6, IL-12p70, CCL2, CCL5, CXCL8 (eBioscience), CCL4, CXCL5, and CXCL10 (R&D systems). Duplicate wells for each donor and condition were averaged for a single value.

### Animals

Female C57BL/6 (CD45.2) and CB6F1 mice (5–7 weeks old), as indicated in each study, were purchased from Charles River Laboratories (Wilmington, MA). B6.SJL-PtprcaPepcb/BoyJ (CD45.1) mice were purchased from Jackson Laboratories (Bar Harbor, Maine). Mice were maintained under specific pathogen-free conditions in the IDRI animal facility and housed in Animal Biological Safety Level-3 (ABSL-3) containment after infection. Unless otherwise indicated, 4 mice per group were included in immunogenicity studies and 7 mice per group were included in challenge studies. Isoflurane was used during non-terminal bleeds in order to minimize animal suffering and distress. All mice in these studies were inspected on a daily basis by the animal technicians familiar with detecting ill animals. The PI, or designated researcher on the project, is responsible for checking the animal if ill, and is required to provide a course of action. Training of new staff was provided by authorized users with monitoring by vivarium staff, following standard operating procedures. After infection of mice with Mtb, mice were closely monitored by the PI or research technicians and euthanized as soon as possible if the indicators such as weight loss, hunched postures, scruffiness of fur, presented themselves. Mice used for long term infection or anticipated to develop fatal TB were weighed by the PIs or technicians just prior to Mtb infection, and at least monthly thereafter. If weight loss exceeding 20% from maximum was noted, mice were euthanized on the same day they reached this endpoint criteria. No mice died prior to meeting the criteria for euthanasia. The total number of animals used in these studies was 264.

### Ethics statement

The IDRI Animal Care and Use Committee (IACUC) approved the protocol for these animal studies. Mice used in these experiments were treated in accordance with the regulations and guidelines of the IACUC (Protocol Numbers: 2011–5, 2014–9, and 2017–9) and with recommendations from the National Institute of Health Guide for the Care and Use of Laboratory Animals. The method of euthanasia used is consistent with the recommendation of the Panel on Euthanasia of the American Veterinary Medical Association. Mice were ethically sacrificed by controlled administration of inhalation carbon dioxide followed by cervical dislocation.

### Immunizations

Mice were immunized intramuscularly with saline or 0.5 μg ID93 admixed with specified adjuvants, at 3-week intervals. The immunizations were administered in 2 sites at 50μl per site for a total of 100 μl; QS21 was used at 2 μg or as noted in the figure legends; GLA was included at 5 μg; ID93 was included at 0.5 μg. Liposomal formulations, provided by the adjuvant team, are described in Table 1.

A subset of CB6F1 mice was immunized intradermally with one dose of bacille Calmette Guérin, Pasteur strain (Sanofi Pasteur, Swiftwater, PA) at 1x10^4^ colony forming units per dose.

### Infection

Four weeks after final immunization, CB6F1 and C57BL/6 mice were challenged with a low dose aerosol (LDA) of *M*. *tuberculosis* HN878 or *M*. *tuberculosis* H37Rv, respectively, using an aerosol exposure chamber (University of Wisconsin, Madison, WI) calibrated to deliver 50–100 colony forming units (CFU) into the lung. Twenty-four hours after infection, lungs of 3 euthanized animals were homogenized and plated on Middlebrook 7H10 agar (Molecular Toxicology, Inc., Boone, NC) to enumerate bacteria delivered. For CB6F1 mice, an average of 79 CFU/lung were delivered; for C57BL/6 mice, an average of 58 CFU/lung were delivered.

### Bacterial burden

Three weeks following challenge with Mtb H37Rv, C57BL/6 mice (7 mice per group) were euthanized to determine bacterial burden in the lungs and spleen. CB6F1 mice were euthanized 4 weeks after challenge with Mtb HN878 for enumeration of bacterial burden. Lung lobes and spleen were homogenized in RPMI using Omni Tissue Homogenizer soft tissue probes (Omni International, Kennesaw, GA). Serial dilutions of homogenates were made in PBS with 0.05% Tween80 (Sigma, St. Louis, MO), and aliquots of dilutions were plated on Middlebrook 7H10 agar plates, with remaining homogenates used for flow cytometry. Plates were incubated for three weeks at 37°C, 5% CO_2_, before colony enumeration. Bacterial burden, in CFU per organ, was calculated and expressed as Log_10_. Reduction in bacterial burden was calculated as (Mean Log_10_ CFUsaline−Mean Log_10_ CFU_vaccine_).

### Flow cytometry

Cells from mouse lung or spleen homogenates were resuspended in RPMI1640 (Life Technologies, Carlsbad, CA)/10%FBS (Sigma) with pen/strep (Life Technologies) and glutamine (Gemini), and dispensed into 96-well round bottom plates. To evaluate cytokine production, cells were stimulated with medium alone, ID93, Rv2608, Rv1813, Rv3619, or Rv3620 at 10 μg/mL, or Mtb lysate (obtained through the Biological and Emerging Infections (BEI) Resources Repository, http://www.beiresources.org) at 1 μg/mL, for 2 hours at 37°C. Subsequently, Brefeldin A at 1 μg/mL (GolgiPlug; BD Biosciences, San Jose, CA) was added and samples incubated an additional 8 hours at 37°C. Plates were held at 4°C overnight before staining with antibodies.

Stimulated cells were incubated with fluorochrome-conjugated monoclonal antibodies to CD4 (clone RM4-5, product # 45-0042-82, eBioscience or 100540, BioLegend, San Diego, CA), CD8 (clone 53–67, product # 100739 or 100752, Biolegend) and CD44 (clone IM7, product # 47-0441-82, eBioscience) in 1% bovine serum albumin (BSA, Sigma) in PBS with 1μg/mL Fc Block (CD16/CD32, Clone 93, product # 14-0161-85, eBioscience) for 10 minutes at room temperature. After washing, cells were treated with Cytofix/Cytoperm (BD Biosciences) for 20 minutes at room temperature, then washed with Perm/Wash buffer (BD Biosciences). Intracellular staining was done with fluorochrome-conjugated monoclonal antibodies to IFNγ (clone XMG1.2, product # 505826), IL-5 (clone TRFK5, product # 504304 or 504306), and IL-17 (clone TC11-18H10.1, product # 506914), all from BioLegend, plus CD154 (clone MR1, product # 12-1541-82 or 46-1541-82), IL-2 (clone JESS-5H4, product # 17-7021-82 or 48-7021-82), and TNF (clone MP6-XT22 product # 11-7321-82, 17-7321-82, or 48-7321-82) from eBioscience in Perm/Wash buffer. All antibodies were used at 1:100 dilution. Stained cells were washed and resuspended in 1% BSA in PBS and filtered before analysis on a modified 4 laser Fortessa with FACSDiva software (BD Biosciences). Lymphocytes were gated by forward and side scatter. Data were analyzed with FlowJo version 10.6.1 (BD Biosciences) and GraphPad Prism 8.1 (BD Biosciences).

### Antibody ELISA

Mice were bled for serum at the time points described in the figure legends. Nunc Polysorp plates (ThermoFisher, Waltham, MA) were coated overnight at 4°C with 2μg/mL of ID93 in 0.1M bicarbonate (ELISA Coating Buffer Powder, eBioscience). Subsequently, plates were blocked with PBS/0.05% Tween20 (TEKnova, Hollister, CA) with 1% BSA for 2h at room temperature (RT). After washing, serial dilutions of serum in PBS/0.05% Tween20/0.1% BSA were added to the plates for 2h at RT. Plates were washed, and HRP-conjugated goat anti-mouse IgA (catalog # 1040–05), IgG1 (catalog # 1070–05), IgG2a (catalog # 1080–05) [21, 22] (performed only in studies done in CB6F1 mice), or IgG2c antibodies (catalog # 1079–05) (performed in studies done in CB6F1 and C57BL/6 mice) (all from Southern Biotech, Birmingham, AL) at 1:2000 dilution were added to the plates, with incubation of 1h at RT. Plates were washed and SureBlue tetramethylbenzidine substrate solution (KPL, Gaithersburg, MD) was added to the plates. After 2 minutes, the reaction was stopped with 1N H_2_SO_4_ and plates were read on a BioTek Synergy2 microplate reader (Winooski, VT) at 450nm with 570nm background subtraction. Reciprocal dilutions corresponding to endpoint titers were determined with GraphPad Prism 8.1 with cutoff value of naïve serum control wells +2SD. Samples with absorbance too low to calculate an endpoint were assigned a value of zero.

### *In vivo* cytotoxicity assay

MHCII+ dependent CD4+ T cell cytotoxicity assay was modified from that previously described [23]. RBC-lysed CD45.1 donor splenocytes were labeled with 0.2μM or 2μM CFSE in PBS for 10 minutes at 37°C, then washed twice with RPMI1640/20% FBS. CFSE^hi^ splenocytes were labeled with 10μM ID93 CD4 peptides at 37°C for 1 hour, then washed. CFSE^lo^ and CFSE^hi^ donor CD45.1 cells were combined 1:1 in PBS, then up to 10^7^ total injected i.v. into immunized CD45.2 recipient mice one week post-immunization, and controls one week post-saline. The next day, splenocytes were harvested from recipients, RBC-lysed, and stained for CD45.1 (clone A20, catalog # 110724), CD45.2 (clone 104, catalog # 109835) and MHC II (I-A^b^ clone AF6-120.1, catalog # 116416) (all BioLegend). Cell gating hierarchy was singlets > lymphocytes > CD45.1^+^ > MHC II^+^ > CFSE^hi^ or CFSE^lo^. The specific lysis was calculated as described previously [24]:

$$[1‐(CFSE^{hi}{}_{immunized}/CFSE^{lo}{}_{immunized})/average(CFSE^{hi}{}_{unimmunized}/CFSE^{lo}{}_{unimmunized})]*100.$$

### Statistical analysis

Human *in vitro* cytokine/chemokine induction was compared between formulations of the same concentration using the Mann-Whitney test, and significance levels (*p < 0.05, **p < 0.01, ***p < 0.001) indicated on graphs. Bacteria burden, single cytokine production after subtraction of medium value, cytokine-polyfunctional T cells, antibody ELISA endpoint titers, and cytotoxicity were assessed versus control groups specified in figures using one-way ANOVA with Bonferroni’s multiple comparison test. Cytokine ELISPOT assays were assessed using 2-way ANOVA with Bonferroni’s multiple comparison test. The above analyses were performed with GraphPad Prism 8.1 (GraphPad Software, San Diego, CA).

## Results

### Selection of the liposomal formulation to pair with synthetic TLR4 agonist, GLA

**Fig 1:**
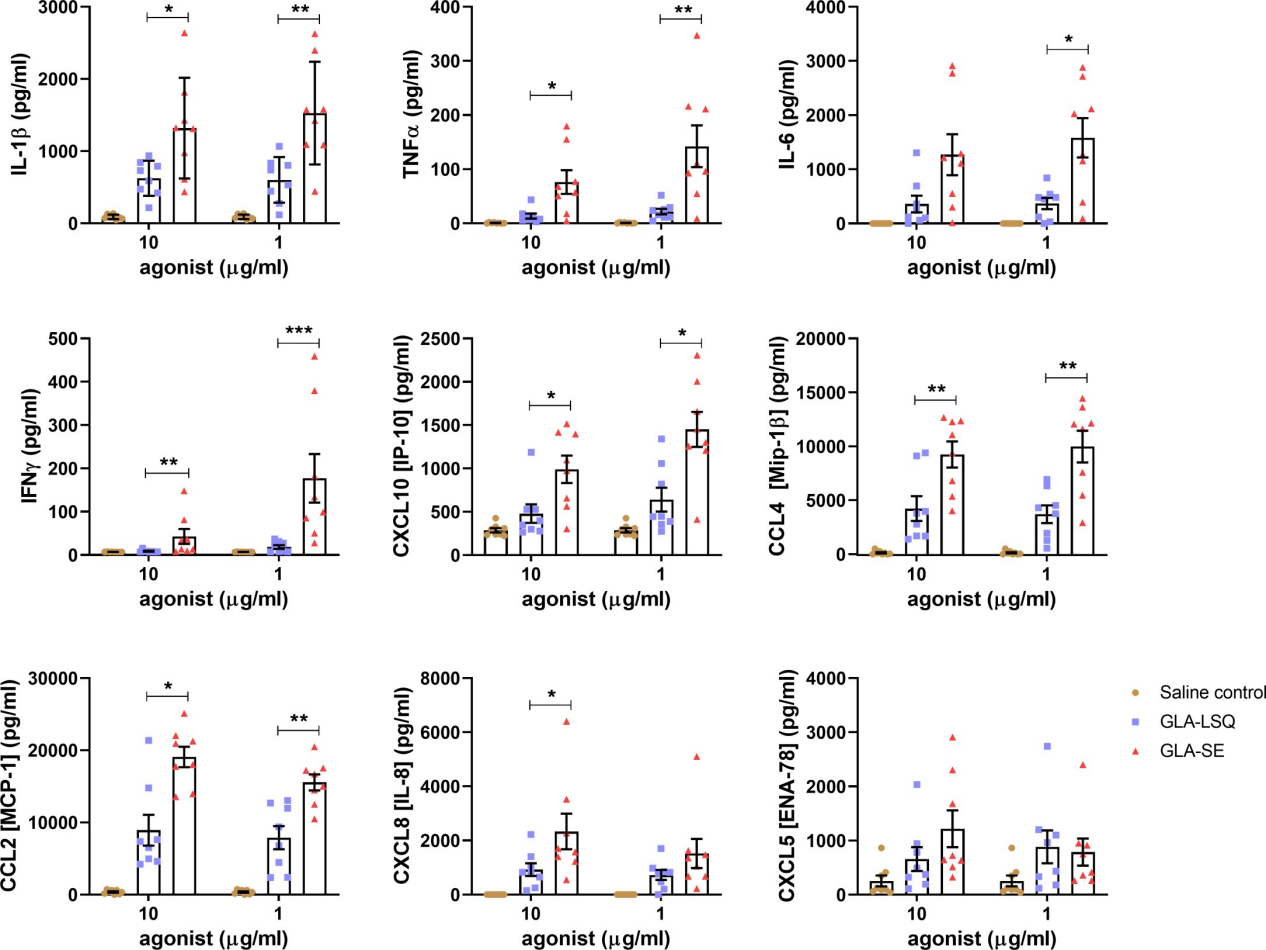
Stimulation of human whole blood with different formulations of GLA. Whole blood from 8 healthy donors was stimulated in duplicate with either 10 or 1 μg/mL of GLA-LSQ, GLA-SE, or saline. Protein levels were measured from the supernatants after 24 hours of stimulation using an ELISA. Duplicates for each donor and condition were averaged for a single value. Data shown are the mean ± SD of those single values for the 8 donors. Statistical significance was determined by the Mann-Whitney test comparing formulations at each concentration, *p<0.05, **p<0.01, ***p<0.001.

**Fig 2:**
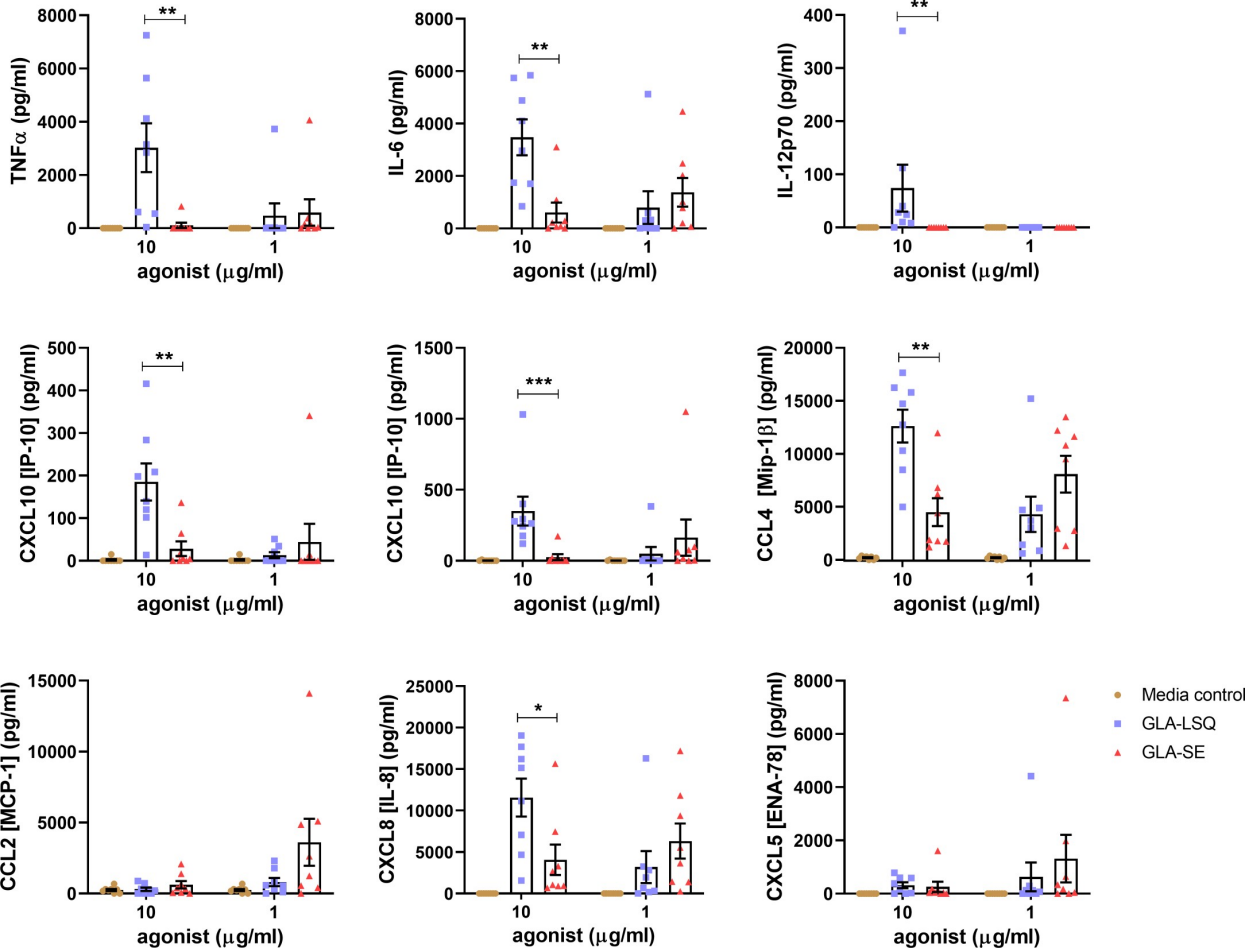
Innate stimulation of human DC with different formulations of GLA. DC prepared from whole blood of 8 human donors were stimulated in duplicate with either 10 or 1 μg/mL of GLA-LSQ, GLA-SE, or media. Protein levels were measured from the supernatants after 24 hours of stimulation using an ELISA. Duplicates for each donor and condition were averaged for a single value. Data shown are the mean ± SD of those single values for the 8 donors. Statistical significance was determined by the Mann-Whitney test comparing formulations at each concentration, *p<0.05, **p<0.01, ***p<0.001.

We evaluated two different formulations of GLA, GLA-LSQ (QS-21+neutral liposomes) and GLA-SE (stable oil-in-water emulsion), for innate responses following their addition to human whole blood (WB) (**Fig 1**) and human dendritic cells (DC) (**Fig 2**). Following WB stimulation with two concentrations of adjuvant (10 and 1 μg/ml), the GLA-SE formulation induced significantly greater levels of innate chemokines and cytokines (including IL-1β, TNF-α, IL-6, IFNγ, CCL2 [MCP-1], CCL4 [MIP-1α], CXCL8 [IL-8], and CXCL10 [IP10] than the GLA-LSQ formulation (**Fig 1**).

In contrast, when human DCs were stimulated with 10 μg/ml of GLA-LSQ and GLA-SE adjuvant formulations, the GLA-LSQ formulation induced significantly greater levels of cytokines and chemokines, including IL-12p70, TNFα, IL-6, CCL4 [Mip-1β], CXCL8 [IL-8], and CXCL10 [IP-10] compared to GLA-SE (**Fig 2**). No significant differences between the formulations were observed at the lower 1 μg/ml concentration.

### In vivo optimization of the liposomes for use in a GLA-LSQ formulation

**Fig 3:**
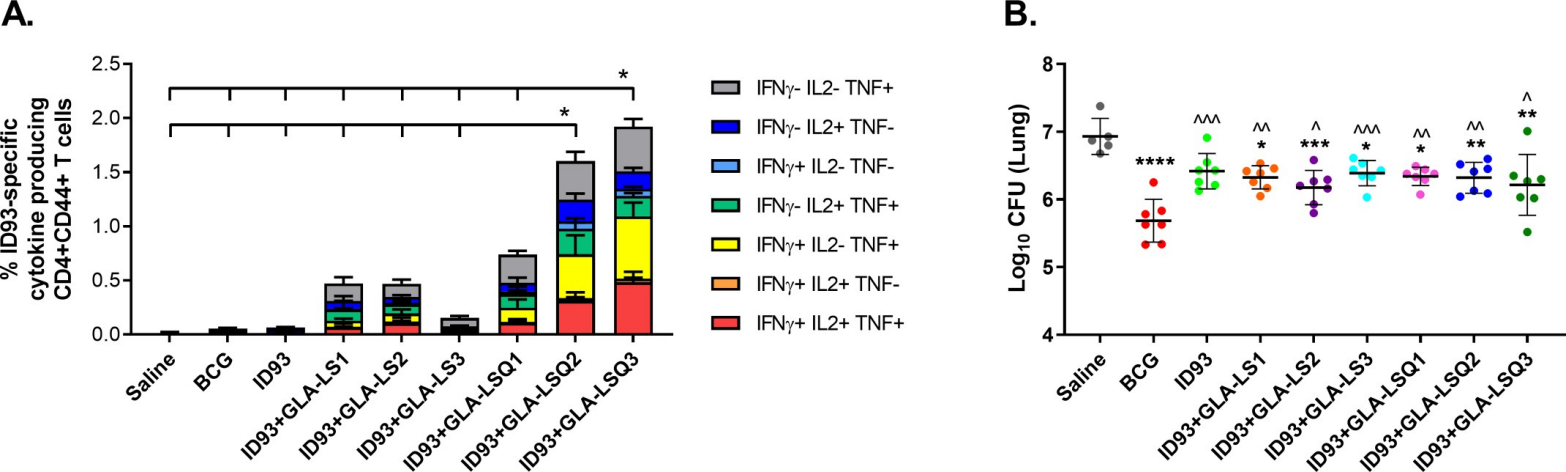
Enhanced protection against Mtb HN878 in CB6F1 mice with liposomal formulations. (A) Increased polyfunctional ID93-specific CD4+CD44+ T cells from the spleens of immunized CB6F1 mice four weeks after the final immunization. The data are represented as the percentage of CD4+CD44+CD154+ T cells producing one or more cytokines following ex-vivo stimulation with ID93; cytokine producing subsets are shown as stacked bars, with mean + SD of each subset. Comparisons of total cytokine producing cells between groups were performed using one-way ANOVA with Bonferroni’s multiple comparison test. Down-inflected lines indicate *p<0.05 versus ID93+GLA-LSQ2 or ID93+GLA-LSQ3. (B) Four weeks after LDA infection with Mtb HN878, lungs were taken, and bacterial burden was determined. Bacterial load within the lung is represented as Log_10_ colony forming units (CFU), with individual mice and mean +/- SD shown. Comparisons among groups were performed using one-way ANOVA with Bonferroni’s multiple comparison test, *p<0.05, **p<0.01, ***p<0.001, ****p<0.0001 versus Saline; ^*p*<0.05, ^^*p*<0.01, ^^^*p*<0.001 versus BCG.

In an effort to evaluate the effect of liposomal composition on GLA adjuvant activity, we tested three liposomal formulations *in vivo*, using ID93 as the vaccine antigen. CB6F1 mice (an F1 cross between C57BL/6 and BALB/c mice) were included for the initial studies in an effort to evaluate responses in mice with a more diverse genetic background in hopes that both cellular-biased responses (C57BL/6) and humoral-biased responses (BALB/c) could be aptly investigated. Mice were immunized three times, three weeks apart, with ID93 combined with three different liposomal formulations of GLA +/- QS-21 (GLA-LS1 [Pegylated], GLA-LS2 [anionic], and GLA-LS3 [neutral]). Spleens were harvested for Immunogenicity four weeks after the last immunization. As shown in **Fig 3A**, the most robust polyfunctional ID93-specific CD4+CD44+CD154+ T cell response, including cells secreting IFNγ, TNF, and IL-2, was observed with ID93+GLA-LSQ2 (anionic) and ID93+GLA-LSQ3 (neutral) candidates. Surprisingly, all of the adjuvanted ID93 vaccines were capable of protecting the CB6F1 mice against the clinical Beijing strain, Mtb HN878 (**Fig 3B**), whereas as expected, the unadjuvanted ID93 fusion protein alone was not protective. BCG, included as a positive control, provided statistically greater protection against Mtb HN878 compared to the ID93 vaccines tested (**Fig 3B**). We next looked at humoral responses induced in immunized mice, where all of the adjuvanted vaccines produced significantly higher antigen-specific IgA, IgG2a, and IgG2c responses compared to ID93 protein (**S1 Fig**). There were no differences in antigen specific IgG1 responses attributed to adjuvant (**S1 Fig**). In addition, all of the adjuvanted ID93 vaccines containing GLA had similar antibody titers regardless of the liposomal formulation (**S1 Fig**). Our next experiments were performed in C57BL/6 mice, in which most of our preclinical work on our TB vaccine candidates have been performed [14, 20, 25, 26]. It was important to compare ID93+GLA-LSQ with ID93 plus the adjuvant that has been most thoroughly tested in the clinic, GLA-SE, therefore the GLA-SE adjuvant was included in subsequent experiments.

### Immunogenicity study to determine the optimal QS-21 dose

**Fig 4:**
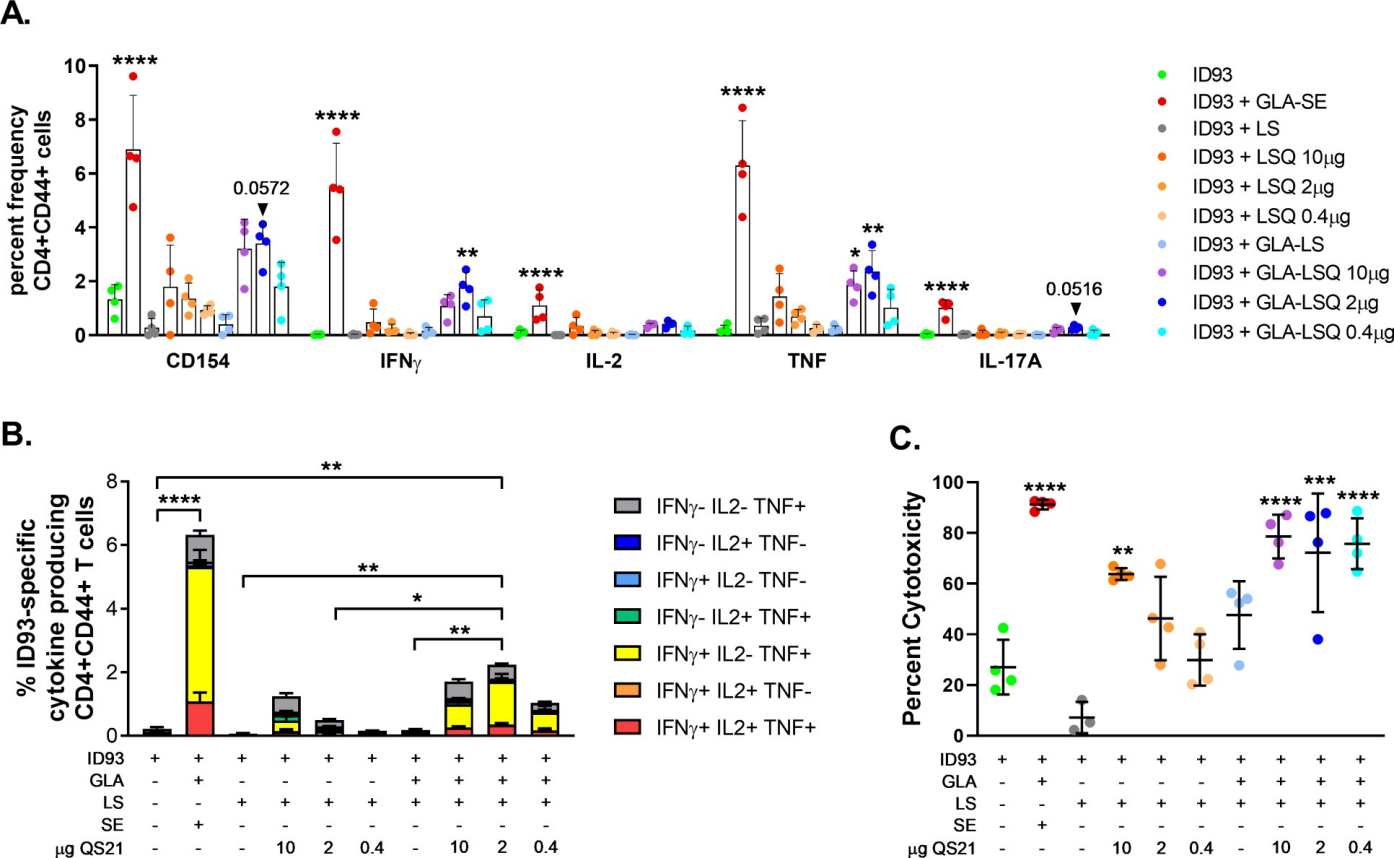
Immunogenicity of C57BL/6 mice four weeks after the final immunization with ID93+GLA-LSQ. Mice were immunized with ID93 combined with GLA-LS containing different QS-21 doses (10, 2 or 0.4 μg). (A) The percent frequency of single-cytokine producing ID93-specific CD4+ T cells. Bars represent the mean of the group, with vertical lines indicating SD. Groups were compared for each cytokine using one-way ANOVA with Bonferroni’s multiple comparison test, with *p<0.05, **p<0.01, ****p<0.0001 versus ID93 alone, an exact p value is shown for values near significance; (B) percent of polyfunctional ID93-specific CD4+CD44+CD154+ T cells producing one or more cytokines. The data are represented as the percentage of CD4+CD44+CD154+ T cells producing one or more cytokines following ex-vivo stimulation with ID93; cytokine producing subsets are shown as stacked bars, with mean + SD of each subset. Comparisons of total cytokine producing cells between groups were performed using one-way ANOVA with Bonferroni’s multiple comparison test, *p<0.05, **p<0.01, ****p<0.0001; (C) percent CD4+ T cell cytotoxicity. Mean and SD, with individual mice, are shown. Comparisons between the groups were performed using one-way ANOVA with Bonferroni’s multiple comparison test; **p<0.01, ***p<0.001, ****p<0.0001 versus ID93 alone.

**Fig 5:**
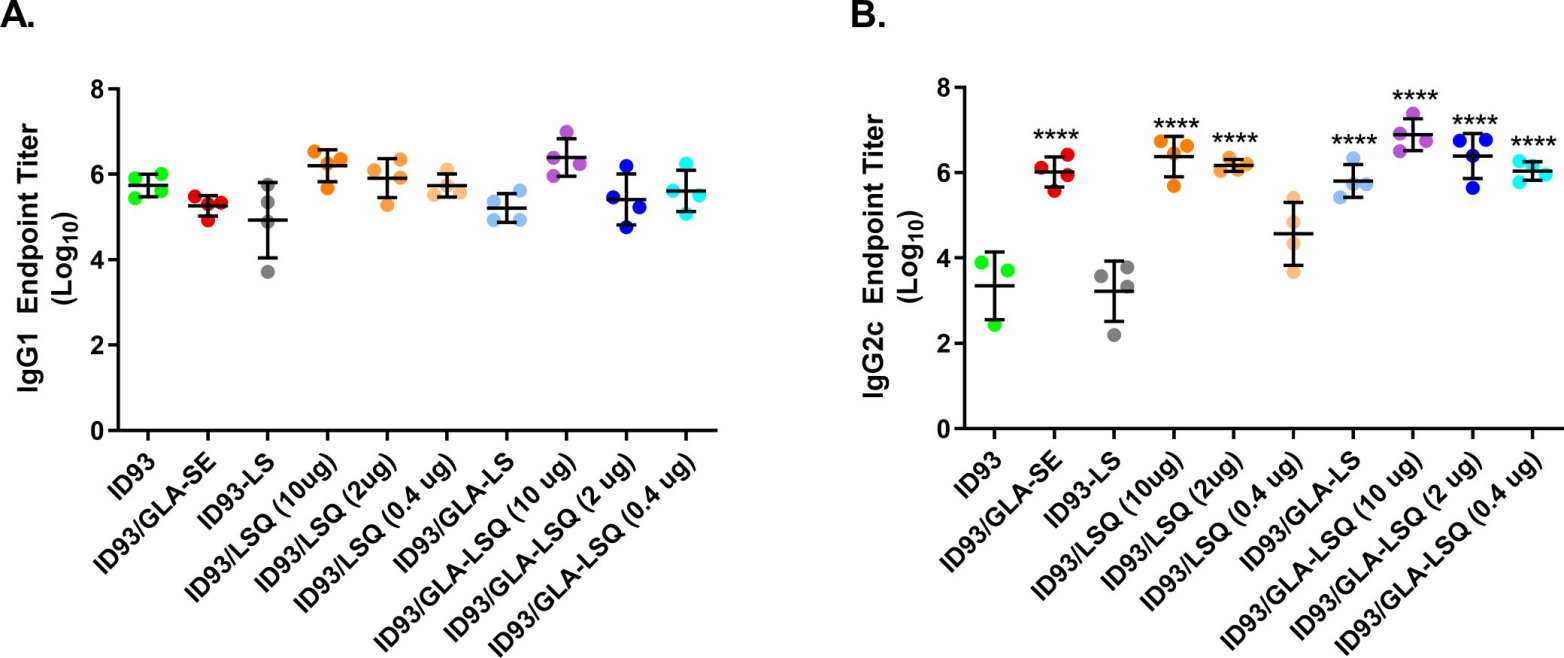
Enhanced ID93-specific IgG2c antibody responses in C57BL/6 mice with ID93+GLA-LSQ. Anti-ID93 IgG1 and IgG2c antibody titers were measured from the sera four weeks after the final immunization using an endpoint antibody ELISA. Results are shown as individual values of 4 mice per group, with mean and SD, and data is representative of two separate experiments. One-way ANOVA with Bonferroni’s multiple comparisons test was used to determine statistical significance of the groups compared to ID93; ****p<0.0001.

Based on the robust T helper 1 (TH1) CD4+ polyfunctional T cell response in addition to the reduction in bacterial load within the lung (**Fig 3**), we moved forward with the neutral formulation LSQ3 for the remainder of the studies. We next wished to determine the effects of different concentrations of QS-21 in the GLA-LSQ liposomal formulation. Our prior study included 2 μg of QS-21 and 5 μg of GLA in the GLA-LSQ formulations. Several different doses of QS-21 in AS01 formulations combined with different vaccines have been used in the mouse model [16]. The M72/AS01_E_ vaccine includes 25 μg MPL and 25 μg of QS-21 in a 0.5 mL dose [4]. Therefore, we performed an immunogenicity study in which three different doses of QS-21, five-fold higher and five-fold lower than the 2 μg dose (10, 2, and 0.4 μg per immunization) were included in the GLA-LSQ adjuvant. The ID93+GLA-SE vaccine candidate was also included as a control to compare the differences in immunity attributed to GLA formulated with an emulsion versus a liposome. C57BL/6 mice were immunized three times, three weeks apart and spleens were harvested from euthanized animals four weeks after the last immunization. As shown in **Fig 4A and 4B**, the ID93 vaccine containing GLA-SE induced the greatest magnitude of CD4+ single cytokine secreting T cells (including IFNγ, TNF, IL-2 and IL-17A) and polyfunctional CD4+ TH1 cells (secreting IFNγ, TNF, and/or IL-2). Increased frequencies of ID93-specific CD4+CD44+ T cells expressing single cytokines (IFNγ, TNF, and IL-2) were also observed in mice given vaccines with adjuvant formulations containing GLA and the 2 μg dose of QS-21. The formulation containing 2 μg of QS-21 also induced significantly greater polyfunctional CD154+CD4+ T cells, whereas neither the 10 nor 0.4 μg QS-21-containing GLA-LSQ formulations were significantly different than the ID93 protein only group (**Fig 4B**). Further results from IFNγ and IL-5 ELISPOT assays showed that a TH1 (IFNγ) response was observed with the ID93+GLA-LSQ (2 μg) immunization, whereas a TH2 (IL-5) response was observed with ID93 protein alone (**S2 Fig**). All of the GLA-LSQ formulations, regardless of QS-21 dose, enhanced cytotoxic capability as measured by *in vivo* lysis of labeled, antigen loaded MHC II+ target cells (**Fig 4C**). ID93-specific CD4+ cytotoxic T cells have previously been reported in ID93+GLA-SE immunized mice [23]. The ID93+GLA-LS (no QS-21) did not induce *in vivo* cytotoxic activity above that of ID93 alone, nor did ID93+LSQ (no GLA) with lower doses of QS-21 at 2 and 0.4 μg. ID93+LSQ containing 10 μg of QS-21, however, did induce a significant cytotoxicity response, although this trended lower than responses induced with GLA-containing adjuvants (**Fig 4C**). Antibody titers were also measured in each of the groups four weeks after the last immunization. ID93-specific IgG1 responses were similar among all groups receiving ID93 regardless of adjuvant formulation or whether the protein was adjuvanted (**Fig 5A**). ID93-specific IgG2c responses were statistically greater than titers in the ID93 protein alone group in all of the groups given ID93+GLA-LSQ regardless of the amount of QS-21 included in the adjuvant (**Fig 5B**), although a dose-dependent trend was observed.

### Side-by-side comparison of efficacy with ID93 combined with different adjuvant formulations

**Fig 6:**
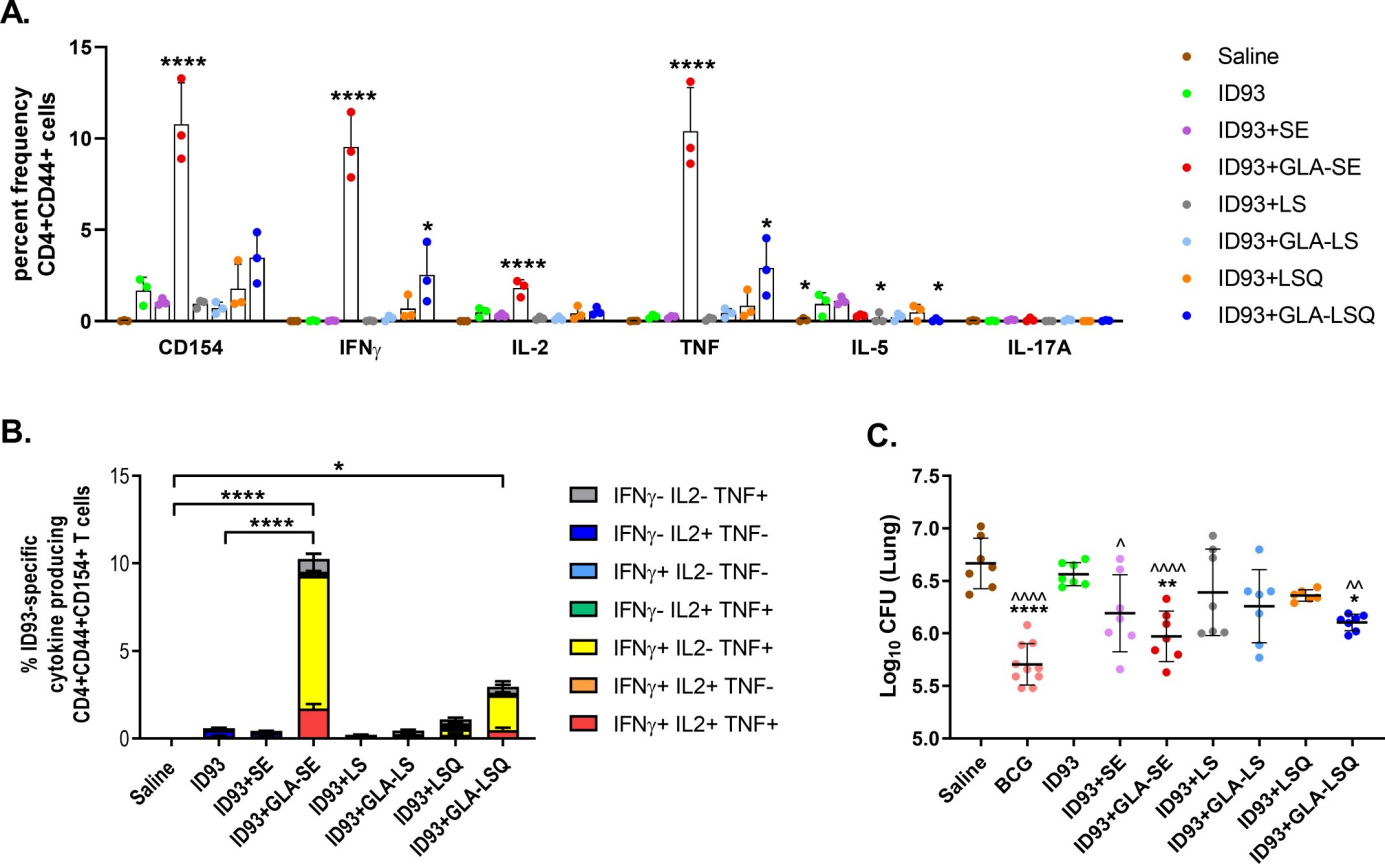
Prophylactic protection with ID93+GLA-SE and ID93+GLA-LSQ in C57BL/6 mice. Mice were immunized with ID93 combined with different adjuvant formulations including SE, LS (liposomes), LSQ (liposomes+QS21), GLA-LS, GLA-LSQ, or GLA-SE. (A) The percent frequency of single-cytokine producing ID93-specific CD4+CD44+ T cells. Bars represent the mean of the group, with vertical line indicating SD. Groups were compared for each cytokine using one-way ANOVA with Bonferroni’s multiple comparison test, with *p<0.05, ****p<0.0001 versus ID93 alone; (B) percent of polyfunctional ID93-specific CD4+CD44+CD154+ T cells producing one or more cytokines; cytokine producing subsets are shown as stacked bars, with mean + SD of each subset. Comparisons between groups were performed using one-way ANOVA with Bonferroni’s multiple comparison test, *p<0.05, ****p<0.0001; (C) bacterial burden in the lung is represented as Log_10_ colony forming units (CFU) 3 weeks after challenge with Mtb H37Rv, with individual mice and mean +/- SD shown. Comparisons between groups were performed using one-way ANOVA with Bonferroni’s multiple comparison test, *p<0.05, **p<0.01, ****p<0.0001 versus Saline; ^*p*<0.05, ^^*p*<0.01, ^^^^*p*<0.0001 versus ID93.

**Fig 7:**
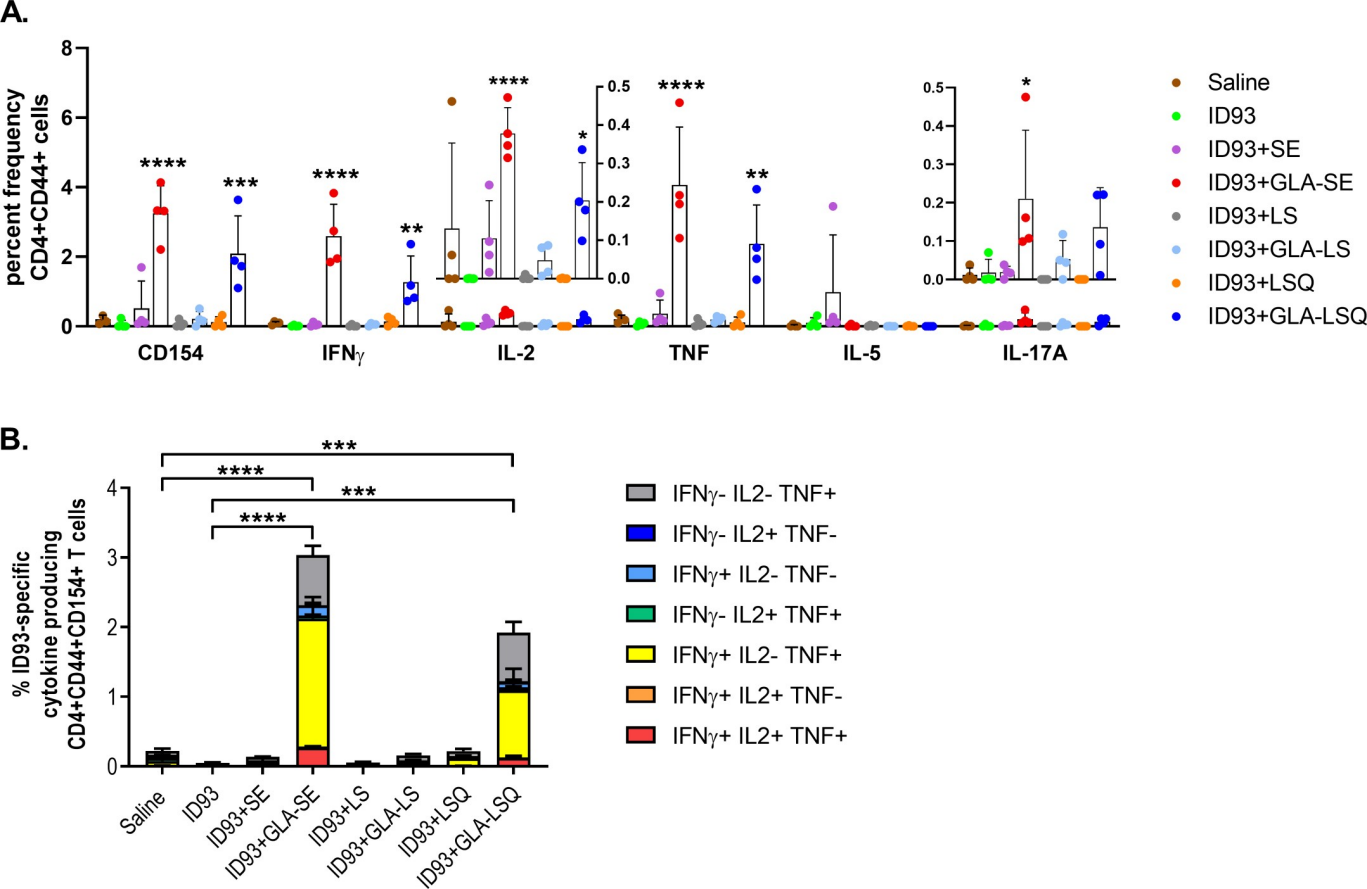
Enhanced TH1 immune responses following infection with Mtb H37Rv within the lungs of C57BL/6 mice immunized with ID93+GLA-SE and ID93+GLA-LSQ. Mice were immunized with ID93 combined with different adjuvant formulations including SE, LS (liposomes), LSQ (liposomes+QS21), GLA-LS, GLA-LSQ, or GLA-SE. Four weeks after the last immunization mice were challenged with a low dose aerosol of Mtb H37Rv. Three weeks after challenge mice were euthanized and lungs collected to assess the immune response. (A) The percent frequency of single-cytokine producing ID93-specific CD4+CD44+ T cells within the lung. Bars represent the mean of the group, with vertical line indicating SD. Groups were compared using one-way ANOVA with Bonferroni’s multiple comparison test, *p<0.05, **p<0.01, ***p<0.001, ****p<0.0001 versus ID93 alone; (B) percent of polyfunctional ID93-specific CD4+CD44+CD154+ T cells within the lung producing one or more cytokines; cytokine producing subsets are shown as stacked bars, with mean + SD of each subset. Comparisons between groups were performed using one-way ANOVA with Bonferroni’s multiple comparison test, ***p<0.001, ****p<0.0001.

Following the immunogenicity results detailed above, a 2μg dose of QS-21 was selected for further evaluation in the GLA-LSQ adjuvant formulation. Having compared the immunogenicity differences between the ID93 vaccines combined with liposomal formulations and ID93+GLA-SE, we set out to perform a side-by-side comparison of the GLA-SE and GLA-LSQ (2 μg QS-21) adjuvant to determine how the adjuvant formulation affects protective efficacy in our mouse model. In addition, we were also interested in determining the contribution of each component of the adjuvant formulations [including the stable emulsion (SE), liposomes alone (LS), and liposomes + QS-21 (LSQ)], therefore we included these adjuvant components combined with ID93 as controls along with ID93+GLA-SE, ID93+GLA-LSQ, and BCG. C57BL/6 mice were immunized once with BCG, or three times, three weeks apart with ID93 protein alone, or ID93 with SE, LS, GLA-SE, GLA-LS, LSQ, or GLA-LSQ, and spleens were harvested four weeks after the last immunization to measure the induced immune responses. The frequency of ID93-specific CD4+CD44+ T cells producing TH1 cytokines, including IFNγ and TNF, was significantly increased compared to ID93 protein alone in the groups that received either ID93+GLA-SE or ID93+GLA-LSQ, however the magnitude of the response was again shown to be greatest with ID93+GLA-SE (**Fig 6A**). In addition, only ID93+GLA-SE induced significant IL-2 responses (**Fig 6A**). Similarly, when comparing polyfunctional CD4+CD44+CD154+ T cell responses, including the production of IFNγ, TNF, and IL-2, the percent frequency of the ID93-specific polyfunctional CD4+ T cell response was significantly higher in the group given ID93+GLA-SE, compared to all other groups (**Fig 6B**). Vaccine efficacy, as demonstrated by a reduction in bacterial counts in the lung, was also determined in the immunized mice. Mice were challenged with a low dose aerosol of Mtb H37Rv four weeks after the last immunization. Three weeks after infection, lungs were harvested from euthanized mice and bacterial burden was assessed in each group. BCG, ID93+GLA-SE and ID93+GLA-LSQ significantly protected against infection compared to both the mock (saline) treated group and the unadjuvanted ID93 groups, although surprisingly, ID93+SE also showed some protection in this study (**Fig 6C**). None of the vaccines combined with individual liposomal components (LS, LSQ, or GLA-LS) elicited protection (**Fig 6C**). Finally, ID93-specific CD4+ TH1 responses were also demonstrated in the lung three weeks after challenge in mice immunized with ID93+GLA-SE and ID93+GLA-LSQ, including significant polyfunctional TH1 responses (**Fig 7**). Interestingly, a minor, yet significant ID93-specific IL-17 response was observed in the lungs of ID93+GLA-SE immunized mice, although the percent frequency of CD4+ T cells secreting IL-17 was lower than the CD4+ IFNγ producing population (**Fig 7A**). Mice immunized with ID93+GLA-LSQ showed a trend towards increased IL-17 in the lung, although the response was not statistically significant (Fig 7A).

## Discussion

The goal of these studies was to optimize a liposomal adjuvant formulation for use with the ID93 vaccine antigen against Mtb. The ID93+GLA-SE vaccine is safe and induces immunity in healthy adult volunteers (Lenexa, Kansas; NCT01599897), in BCG-vaccinated healthy adults (South Africa; NCT0192719), and when given at the end of drug treatment in HIV-uninfected TB patients (South Africa; NCT02465216) [10, 12].

In this work, we have characterized the GLA-LSQ adjuvant formulation (which contains a synthetic TLR4 agonist plus QS-21 in a liposomal formulation; this is similar to AS01 which instead contains MPL and QS-21 in a liposomal formulation) for prophylactic TB vaccine efficacy in the mouse model. The differences between the synthetic (hexaacylated lipid A derivative; GLA) and natural (purified non-toxic derivative of LPS derived from *Salmonella minnesota* R595; MPL) TLR4 agonists have previously been described [8]. Adjuvants such as MPL and GLA help to drive adaptive immunity by enhancing antigen presentation (including upregulation of MHC molecules), DC maturation, and stimulation of innate cytokines and chemokines associated with immune cell trafficking [8]. It is interesting to highlight that GLA consists of a single highly pure hexaacylated structure whereas MPL consists of a mixture of structures with different numbers of acyl chains including a hexaacylated form which is considered to be the most active structure for activation of human cells [27]. Although both MPL and GLA have demonstrated acceptable safety profiles in clinical testing, this compositional difference may explain why GLA is employed in clinical testing at approximately one order of magnitude lower doses than MPL.

We first characterized cytokine and chemokine responses on human WB and DCs upon stimulation with two different formulations of GLA; one formulated with a stable oil-in-water emulsion (SE) and the other formulated with a QS-21 containing liposomal formulation (similar to AS01). Stimulation of human whole blood (WB) resulted in a higher magnitude of cytokine/chemokine responses with GLA-SE, whereas stimulation of human DCs led to significantly higher cytokine/chemokine responses with the GLA-LSQ formulation over the GLA-SE adjuvant. This data is in line with the AS01 adjuvant system, which has been shown to require MHCII^high^ DC’s for priming optimal adaptive T cell responses [28]. We have previously reported gene expression from both mouse and human DCs in response to GLA (and MPL) in an aqueous suspension, which led to upregulation and secretion of IL-6, TNF, CCL4, IL-12, and CXCL10 [8], similar to what was observed on human DC stimulated with GLA-LSQ in the current study. IFNγ was observed following stimulation of human WB with GLA-SE. Adaptive TH1 immunity following immunization with ID93+GLA-SE has been shown to depend on IL-12 and T-bet in mice, and innate IFNγ from NK and CD8+ T cells is dependent on type 1 IFN [29]. Work done by Dubois Cauwelaert et al. showed that early after immunization in mice, both NK and CD8+ T cells produce IFNγ which is significantly reduced when type 1 IFN signaling is blocked [29]. Here we show that IFNγ-inducible CXCL10 (IFNγ-induced-protein-10) was produced following WB stimulation with GLA-SE and is also produced following stimulation with GLA-LSQ from human DC.

We have also previously demonstrated that GLA-SE combined with ID93 stimulates a potent TH1 CD4+ T cell response [14, 25, 26, 30]. The AS01 adjuvant in combination with several different vaccines also results in strong TH1 and humoral immunity [16, 31–33]. In this study, we wanted to determine whether ID93 combined with an optimized GLA-LSQ formulation could induce TH1 CD4+ T cell responses and provide protection in vivo in our preclinical mouse model. We initially tested ID93 with different liposomal formulations in CB6F1 mice. The LSQ formulations that induced the greatest percent frequency of ID93-specific polyfunctional TH1 responses (CD4+CD44+CD154+ T cells expressing IFNγ, TNF, and/or IL-2) were the neutral liposome (GLA-LSQ3), followed closely by the anionic liposome (GLA-LSQ2) formulation. Having shown that the GLA-LSQ formulations had the highest TH1 immune responses, we were surprised to see that all of the liposomal formulations regardless of whether they include QS-21 or not were capable of providing protection in the CB6F1 mouse model. Interestingly, all of the adjuvanted formulations induced significant antigen specific IgG2a and IgG2c antibody titers (in addition to IgA responses). In humans, highly functional antibody subclass responses (IgG1 and IgG3) are induced following three immunizations with ID93+GLA-SE, resulting in enhanced NK ADCC responses shown by the production of IFNγ and MIP1β, and upregulation of CD107a [10]. Antibody-dependent cellular phagocytosis (ADCP) is also significantly increased in humans immunized with ID93+GLA-SE, but not antigen alone [10]. Lu et al. have shown that individuals with latent TB have superior PPD-specific antibodies capable of driving ADCC and NK cell activation compared to individuals with active TB [34]. We speculate that perhaps the enhanced ID93-specific IgG2 responses in our mouse study were partially responsible for the enhanced protection against Mtb in the CB6F1 mouse model, which is something that we are interested in pursuing.

In the next set of experiments, we wished to include ID93+GLA-SE given as a prophylactic vaccine to enable a side-by-side comparison with the ID93 vaccine combined with an ‘ASO1-like’ adjuvant (GLA + QS-21 in liposomes) in the C57BL/6 model. The M72 tuberculosis POD vaccine candidate combined with AS01 has shown promise in humans against Mtb, where 54% protection against disease has been reported [4]. Furthermore, the AS01 adjuvant is included in the FDA approved Shingrix vaccine, which is highly effective (97%) in older adults against shingles, and in the EMA recommended RTS,S malaria vaccine (Mosquirix) [35]. We selected the neutral liposomal GLA-LSQ formulation; the anionic GLA-LSQ formulation could be an alternate candidate for development based on the enhanced the magnitude of the CD4+ TH1 polyfunctional responses and protection that was observed with both formulations. In order to determine the optimal concentration of QS-21, three concentrations of QS-21 were tested (10, 2, and 0.4 μg) which were combined with liposomes, or liposome and GLA; ID93+GLA-SE was included as a control. TH1 responses were induced with ID93+GLA-SE and with ID93+GLA-LSQ containing the 2 μg dose of QS-21. A small, but significant ID93-specific IL-17 response was also observed with ID93+ GLA-SE. A significant percent of CD4+ T cell cytotoxicity was observed with ID93+GLA-SE as previously shown [23]. Interestingly, while there was a clear dose response for CD4+ cytotoxicity in mice given ID93+LSQ (without GLA, only the high dose of QS-21 was considered significant), combining GLA with LSQ enhanced cytotoxic responses to nearly equal levels regardless of the QS-21 dose. We also measured humoral responses, which may contribute to immunity against Mtb [10, 34, 36–38]. All of the GLA-containing adjuvants induced ID93-specific IgG2c and IgA serum antibody responses above that seen with protein only. Interestingly, whereas immunization with ID93 combined with the liposomal formulation (without QS-21 or GLA) did not lead to induction of ID93-specific IgG2c responses compared to protein alone, the LSQ formulation (without GLA), and the liposomal formulation (with GLA), in addition to the GLA-LSQ adjuvants did significantly increase ID93-specific IgG2c responses. The addition of GLA to liposomes with the lowest amount of QS-21 (0.4 μg) also led to a significant increase in ID93-specific IgG2c. AS01 also results in the production of polyfunctional CD4 T cells in addition to a TH1-isotype switching humoral response [31]. Studies done in T-bet and IL-12 knockout mice indicate that ID93-specific IgG2c production following ID93+GLA-SE immunization is dependent on T-bet but not IL-12, even though IL-12 is required for adaptive ID93-specific TH1 responses [29]. Based on both *in vitro* and *in vivo* responses, we selected the neutral formulation for further studies.

In our next set of studies, we further defined the effects of the liposomal adjuvant formulations using 2 μg QS-21. We measured both immunogenicity and protective efficacy with ID93+GLA-SE compared to ID93+GLA-LSQ, liposomes alone, and LSQ without the TLR agonist. ID93 combined with either GLA-SE or GLA-LSQ induced CD4+ TH1 cells producing IFNγ and TNF, however ID93 formulated with the GLA-SE-containing adjuvant induced significantly greater IL-2 and ID93-specific polyfunctional TH1 cells than the GLA-LSQ adjuvant. Although GLA-SE induced a more robust TH1 immune response versus GLA-LSQ, both vaccines were able to provide protective efficacy in C57BL/6 mice when given as a prophylactic vaccine compared to the ID93 protein alone, following a low dose aerosol challenge with Mtb H37Rv. This suggests that the adjuvants may be working through different mechanisms, which have not yet been fully characterized, or alternatively that the magnitude of the TH1 response is not critical to protection against Mtb. When immune responses were examined within the lung following Mtb challenge, enhanced TH1 responses were observed, including ID93-specific IFNγ, TNF, and IL-2 with both ID93+GLA-SE and ID93+GLA-LSQ. In addition, a significant ID93-specific IL-17 response was induced post infection with ID93+GLA-SE, and trended slightly higher with ID93+GLA-LSQ. There has been recent interest in the generation of both a TH1 and TH17 immune response against Mtb. We show that this is achievable in mice with GLA-SE combined with ID93 when given intramuscularly. The contribution of antigen-specific CD4+ T cells expressing CXCR3 and CCR6 along with production of both IFNγ and IL-17 may be indicative of protective responses against Mtb, as shown in a rhesus macaque model of latent tuberculosis [39]. Our group is currently investigating the kinetics of lung homing T cells and phenotypes following immunization with ID93 formulated with different adjuvants to determine whether cells from vaccinated animals more effectively localize to specific areas of the lung, such as close proximity to granulomas.

## Conclusions

This work characterizes liposomal formulations containing the TLR4 agonist (GLA) and QS-21 in combination with ID93, for use as a vaccine against Mtb. We predominantly show an ID93-specific TH1 cellular immune response, including CD4+ T cell production of IFNγ, TNF, and IL-2 in mice immunized with both ID93+GLA-SE and GLA-LSQ, in addition to vaccine-specific IgG2 humoral responses. Both ID93+GLA-SE and ID93+GLA-LSQ also reduced the bacterial load in the lungs of mice infected with Mtb. Looking forward, it will be of interest to determine if these preclinical mouse results translate to those in humans. A study in healthy adults evaluating the safety, tolerability, and immunogenicity of a liposomal formulation combined with ID93 [ID93+AP10-602 (GLA-LSQ)], ID93+GLA-SE, or ID93 alone, has been completed and results are pending (Iowa City, Iowa; NCT02508376). In this study, two doses (5 and 10 μg GLA) of AP10-602 combined with ID93 (10 μg) were compared to 5 μg of GLA-SE combined with ID93 (10 μg), or ID93 (10 μg) alone. The availability of banked biospecimens and ongoing correlate of protection studies from human clinical trials, including the M72/AS01 POD trial, will also provide invaluable data that will further enable the development of late stage TB vaccines [40] including the ID93+AP10-602 vaccine. Given limited resources, early correlative biomarkers of protection could reduce the costs of human clinical trials and advance highly effective TB vaccine candidates in the pipeline.

## Supporting information

S1 Fig Anti-ID93 antibody responses in the CB6F1 mouse model. Enhanced ID93-specific IgA, and IgG2a and IgG2c antibody responses were observed with ID93 (no adjuvant) compared to saline. All GLA-containing adjuvants combined with ID93 induced higher ID93-specific IgA, IgG2a, and IgG2c antibody responses compared to ID93 alone, 4 weeks after the last immunization. Results are shown as the individual values of 4 mice per group, with average and SD. One-way ANOVA with Bonferroni’s multiple comparisons test was used to determine statistical significance among groups, indicated by horizontal bars; ***p<0.001, ****p<0.0001.
(TIF)

S2 Fig Decreased Th2 immunity with ID93+GLA-LSQ compared to ID93 alone. C57BL/6 mice were immunized three times, three weeks apart with ID93 or ID93+GLA-LSQ. Four weeks after the last immunization, spleens were harvested from 4 mice per group, and stimulated with ID93 (10 μg/mL), an ID93 CD4 peptide pool (1 μg/mL) or CD8 peptide pool (1 μg/mL). An (A) IFNγ or (B) IL-5 ELISPOT was performed as previously described [25]. Comparisons were performed using a 2-way ANOVA of ID93 versus ID93+GLA-LSQ for each stimulation, and medium versus stimulations for each immunization, with Bonferroni’s multiple comparison test, **p < 0.01, ****p < 0.0001.
(TIF)

## References

[1] Abubakar I, Pimpin L, Ariti C, Beynon R, Mangtani P, Sterne JA, et al. Systematic review and meta-analysis of the current evidence on the duration of protection by bacillus Calmette-Guerin vaccination against tuberculosis. Health Technol Assess. 2013;17(37):1–372, v-vi. 10.3310/hta17370 24021245 PMC4781620

[2] Hesseling AC, Johnson LF, Jaspan H, Cotton MF, Whitelaw A, Schaaf HS, et al. Disseminated bacille Calmette-Guerin disease in HIV-infected South African infants. Bull World Health Organ. 2009;87(7):505–11. 10.2471/blt.08.055657 19649364 PMC2704039

[3] Talbot EA, Perkins MD, Silva SF, Frothingham R. Disseminated bacille Calmette-Guerin disease after vaccination: case report and review. Clin Infect Dis. 1997;24(6):1139–46. 10.1086/513642 9195072

[4] Van Der Meeren O, Hatherill M, Nduba V, Wilkinson RJ, Muyoyeta M, Van Brakel E, et al. Phase 2b Controlled Trial of M72/AS01E Vaccine to Prevent Tuberculosis. N Engl J Med. 2018;379(17):1621–34. 10.1056/NEJMoa1803484 30280651 PMC6151253

[5] Tait DR, Hatherill M, Van Der Meeren O, Ginsberg AM, Van Brakel E, Salaun B, et al. Final Analysis of a Trial of M72/AS01E Vaccine to Prevent Tuberculosis. N Engl J Med. 2019;381(25):2429–39. 10.1056/NEJMoa1909953 31661198

[6] Barnes VL, Fedor DM, Williams S, Dowling QM, Archer MC, Cloutier S, et al. Lyophilization of an Adjuvanted Mycobacterium tuberculosis Vaccine in a Single-Chamber Pharmaceutical Cartridge. AAPS PharmSciTech. 2017;18(6):2077–84. 10.1208/s12249-016-0688-7 28000085

[7] Orr MT, Kramer RM, Barnes Lt, Dowling QM, Desbien AL, Beebe EA, et al. Elimination of the cold-chain dependence of a nanoemulsion adjuvanted vaccine against tuberculosis by lyophilization. J Control Release. 2014;177:20–6. 10.1016/j.jconrel.2013.12.025 24382398 PMC3956454

[8] Coler RN, Bertholet S, Moutaftsi M, Guderian JA, Windish HP, Baldwin SL, et al. Development and characterization of synthetic glucopyranosyl lipid adjuvant system as a vaccine adjuvant. PLoS One. 2011;6(1):e16333. 10.1371/journal.pone.0016333 21298114 PMC3027669

[9] Carter D, van Hoeven N, Baldwin S, Levin Y, Kochba E, Magill A, et al. The adjuvant GLA-AF enhances human intradermal vaccine responses. Sci Adv. 2018;4(9):eaas9930. 10.1126/sciadv.aas9930 30221194 PMC6136895

[10] Coler RN, Day TA, Ellis R, Piazza FM, Beckmann AM, Vergara J, et al. The TLR-4 agonist adjuvant, GLA-SE, improves magnitude and quality of immune responses elicited by the ID93 tuberculosis vaccine: first-in-human trial. NPJ Vaccines. 2018;3:34. 10.1038/s41541-018-0057-5 30210819 PMC6123489

[11] Mordmuller B, Sulyok M, Egger-Adam D, Resende M, de Jongh WA, Jensen MH, et al. First-in-human, Randomized, Double-blind Clinical Trial of Differentially Adjuvanted PAMVAC, A Vaccine Candidate to Prevent Pregnancy-associated Malaria. Clin Infect Dis. 2019;69(9):1509–16. 10.1093/cid/ciy1140 30629148 PMC6792113

[12] Penn-Nicholson A, Tameris M, Smit E, Day TA, Musvosvi M, Jayashankar L, et al. Safety and immunogenicity of the novel tuberculosis vaccine ID93 + GLA-SE in BCG-vaccinated healthy adults in South Africa: a randomised, double-blind, placebo-controlled phase 1 trial. Lancet Respir Med. 2018;6(4):287–98. 10.1016/S2213-2600(18)30077-8 29595510

[13] Santini-Oliveira M, Coler RN, Parra J, Veloso V, Jayashankar L, Pinto PM, et al. Schistosomiasis vaccine candidate Sm14/GLA-SE: Phase 1 safety and immunogenicity clinical trial in healthy, male adults. Vaccine. 2016;34(4):586–94. 10.1016/j.vaccine.2015.10.027 26571311

[14] Bertholet S, Ireton GC, Ordway DJ, Windish HP, Pine SO, Kahn M, et al. A defined tuberculosis vaccine candidate boosts BCG and protects against multidrug-resistant Mycobacterium tuberculosis. Sci Transl Med. 2010;2(53):53ra74. 10.1126/scitranslmed.3001094 20944089 PMC3110937

[15] Day CL, Tameris M, Mansoor N, van Rooyen M, de Kock M, Geldenhuys H, et al. Induction and regulation of T-cell immunity by the novel tuberculosis vaccine M72/AS01 in South African adults. Am J Respir Crit Care Med. 2013;188(4):492–502. 10.1164/rccm.201208-1385OC 23306546 PMC3778736

[16] Lacaille-Dubois MA. Updated insights into the mechanism of action and clinical profile of the immunoadjuvant QS-21: A review. Phytomedicine. 2019;60:152905. 10.1016/j.phymed.2019.152905 31182297 PMC7127804

[17] Alving CR, Peachman KK, Rao M, Reed SG. Adjuvants for human vaccines. Curr Opin Immunol. 2012;24(3):310–5. 10.1016/j.coi.2012.03.008 22521140 PMC3383374

[18] Misquith A, Fung HW, Dowling QM, Guderian JA, Vedvick TS, Fox CB. In vitro evaluation of TLR4 agonist activity: formulation effects. Colloids Surf B Biointerfaces. 2014;113:312–9. 10.1016/j.colsurfb.2013.09.006 24121074 PMC3877169

[19] Qi Y, Fox CB. A Two-Step Orthogonal Chromatographic Process for Purifying the Molecular Adjuvant QS-21 with High Purity and Yield. J Chromatogr A. 2021;1635:461705. 10.1016/j.chroma.2020.461705 33234294 PMC7770036

[20] Orr MT, Fox CB, Baldwin SL, Sivananthan SJ, Lucas E, Lin S, et al. Adjuvant formulation structure and composition are critical for the development of an effective vaccine against tuberculosis. J Control Release. 2013;172(1):190–200. 10.1016/j.jconrel.2013.07.030 23933525 PMC3871206

[21] Jouvin-Marche E, Morgado MG, Leguern C, Voegtle D, Bonhomme F, Cazenave PA. The mouse Igh-1a and Igh-1b H chain constant regions are derived from two distinct isotypic genes. Immunogenetics. 1989;29(2):92–7. 10.1007/BF00395856 2563358

[22] Martin RM, Brady JL, Lew AM. The need for IgG2c specific antiserum when isotyping antibodies from C57BL/6 and NOD mice. J Immunol Methods. 1998;212(2):187–92. 10.1016/s0022-1759(98)00015-5 9672206

[23] Coler RN, Hudson T, Hughes S, Huang PW, Beebe EA, Orr MT. Vaccination Produces CD4 T Cells with a Novel CD154-CD40-Dependent Cytolytic Mechanism. J Immunol. 2015;195(7):3190–7. 10.4049/jimmunol.1501118 26297758 PMC4575887

[24] Jellison ER, Kim SK, Welsh RM. Cutting edge: MHC class II-restricted killing in vivo during viral infection. J Immunol. 2005;174(2):614–8. 10.4049/jimmunol.174.2.614 15634878

[25] Baldwin SL, Bertholet S, Reese VA, Ching LK, Reed SG, Coler RN. The importance of adjuvant formulation in the development of a tuberculosis vaccine. J Immunol. 2012;188(5):2189–97. 10.4049/jimmunol.1102696 22291184 PMC3288309

[26] Baldwin SL, Reese VA, Huang PW, Beebe EA, Podell BK, Reed SG, et al. Protection and Long-Lived Immunity Induced by the ID93/GLA-SE Vaccine Candidate against a Clinical Mycobacterium tuberculosis Isolate. Clin Vaccine Immunol. 2016;23(2):137–47. 10.1128/CVI.00458-15 26656121 PMC4744918

[27] Fox DC C.B., Kramer R.M., Beckman A.M., Reed S.G. Current Status of Toll-like Receptor 4 Ligand Vaccine Adjuvants. Immunopotentiators in Modern Vaccines. Second Edition ed: Elsevier Ltd.; 2017. p. 105–27.

[28] Didierlaurent AM, Collignon C, Bourguignon P, Wouters S, Fierens K, Fochesato M, et al. Enhancement of adaptive immunity by the human vaccine adjuvant AS01 depends on activated dendritic cells. J Immunol. 2014;193(4):1920–30. 10.4049/jimmunol.1400948 25024381

[29] Dubois Cauwelaert N, Desbien AL, Hudson TE, Pine SO, Reed SG, Coler RN, et al. The TLR4 Agonist Vaccine Adjuvant, GLA-SE, Requires Canonical and Atypical Mechanisms of Action for TH1 Induction. PLoS One. 2016;11(1):e0146372. 10.1371/journal.pone.0146372 26731269 PMC4701231

[30] Kwon KW, Lee A, Larsen SE, Baldwin SL, Coler RN, Reed SG, et al. Long-term protective efficacy with a BCG-prime ID93/GLA-SE boost regimen against the hyper-virulent Mycobacterium tuberculosis strain K in a mouse model. Sci Rep. 2019;9(1):15560. 10.1038/s41598-019-52146-0 31664157 PMC6820558

[31] Coccia M, Collignon C, Herve C, Chalon A, Welsby I, Detienne S, et al. Cellular and molecular synergy in AS01-adjuvanted vaccines results in an early IFNgamma response promoting vaccine immunogenicity. NPJ Vaccines. 2017;2:25. 10.1038/s41541-017-0027-3 29263880 PMC5627273

[32] Didierlaurent AM, Laupeze B, Di Pasquale A, Hergli N, Collignon C, Garcon N. Adjuvant system AS01: helping to overcome the challenges of modern vaccines. Expert Rev Vaccines. 2017;16(1):55–63. 10.1080/14760584.2016.1213632 27448771

[33] van den Berg RA, De Mot L, Leroux-Roels G, Bechtold V, Clement F, Coccia M, et al. Adjuvant-Associated Peripheral Blood mRNA Profiles and Kinetics Induced by the Adjuvanted Recombinant Protein Candidate Tuberculosis Vaccine M72/AS01 in Bacillus Calmette-Guerin-Vaccinated Adults. Front Immunol. 2018;9:564. 10.3389/fimmu.2018.00564 29632533 PMC5879450

[34] Lu LL, Chung AW, Rosebrock TR, Ghebremichael M, Yu WH, Grace PS, et al. A Functional Role for Antibodies in Tuberculosis. Cell. 2016;167(2):433–43 e14. 10.1016/j.cell.2016.08.072 27667685 PMC5526202

[35] Morrison C. Landmark green light for Mosquirix malaria vaccine. Nat Biotechnol. 2015;33(10):1015–6. 10.1038/nbt1015-1015 26448075

[36] Achkar JM, Chan J, Casadevall A. B cells and antibodies in the defense against Mycobacterium tuberculosis infection. Immunol Rev. 2015;264(1):167–81. 10.1111/imr.12276 25703559 PMC4629253

[37] Chan J, Mehta S, Bharrhan S, Chen Y, Achkar JM, Casadevall A, et al. The role of B cells and humoral immunity in Mycobacterium tuberculosis infection. Semin Immunol. 2014;26(6):588–600. 10.1016/j.smim.2014.10.005 25458990 PMC4314354

[38] Choreno-Parra JA, Weinstein LI, Yunis EJ, Zuniga J, Hernandez-Pando R. Thinking Outside the Box: Innate- and B Cell-Memory Responses as Novel Protective Mechanisms Against Tuberculosis. Front Immunol. 2020;11:226. 10.3389/fimmu.2020.00226 32117325 PMC7034257

[39] Shanmugasundaram U, Bucsan AN, Ganatra SR, Ibegbu C, Quezada M, Blair RV, et al. Pulmonary Mycobacterium tuberculosis control associates with CXCR3- and CCR6-expressing antigen-specific Th1 and Th17 cell recruitment. JCI Insight. 2020;5(14). 10.1172/jci.insight.137858 32554933 PMC7453885

[40] Ginsberg AM. Designing tuberculosis vaccine efficacy trials—lessons from recent studies. Expert Rev Vaccines. 2019;18(5):423–32. 10.1080/14760584.2019.1593143 30892969 PMC6492041

