# Supplementary figures and images for: Prophylactic efficacy against Mycobacterium tuberculosis using ID93 and lipid-based adjuvant formulations in the mouse model
Source: PLoS One. 2021 Mar 11;16(3):e0247990. doi: 10.1371/journal.pone.0247990 (PMC7951850; doi:10.1371/journal.pone.0247990)

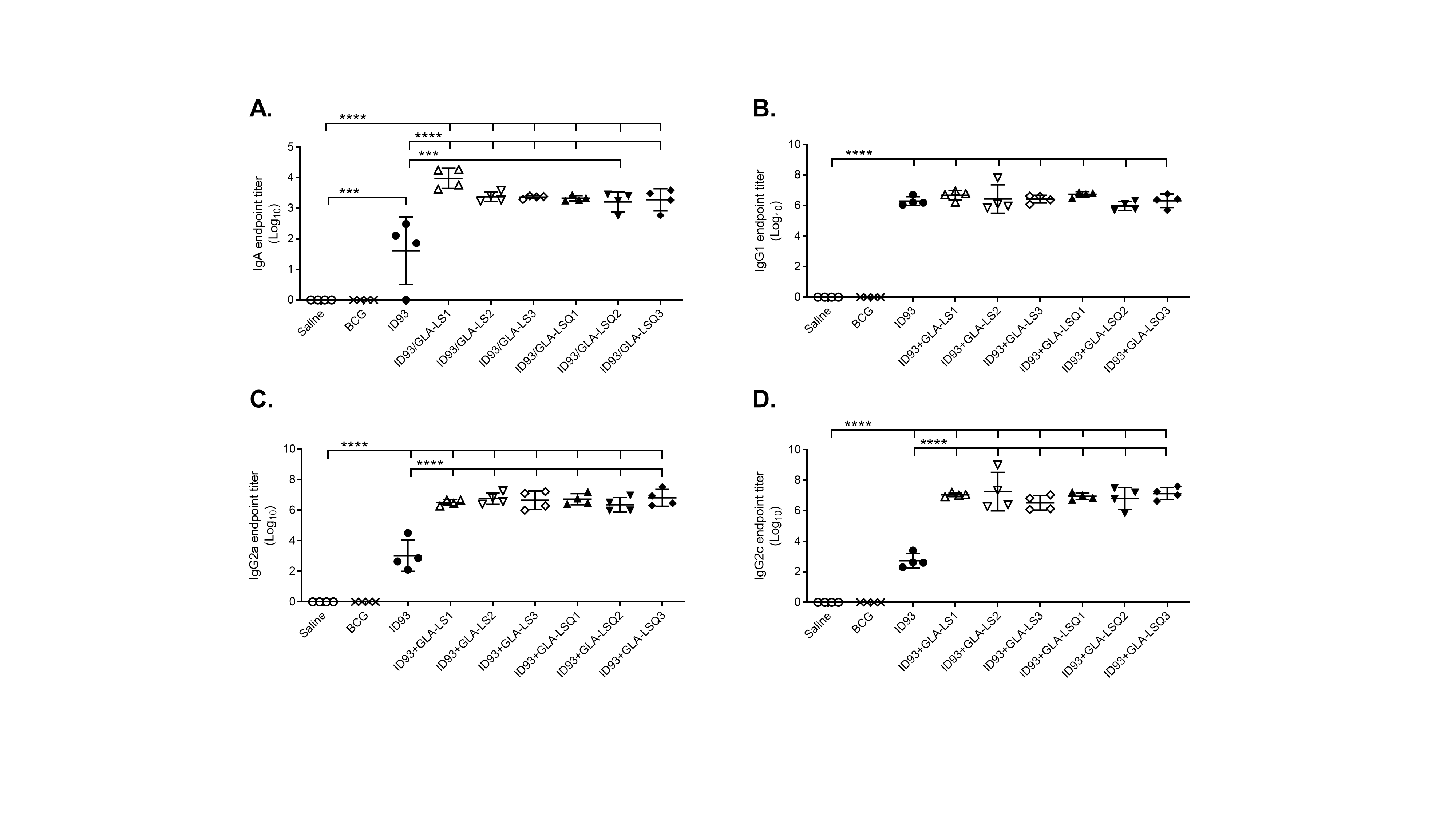

Supplement: S1 Fig — Enhanced ID93-specific IgA, and IgG2a and IgG2c antibody responses were observed with ID93 (no adjuvant) compared to saline. All GLA-containing adjuvants combined with ID93 induced higher ID93-specific IgA, IgG2a, and IgG2c antibody responses compared to ID93 alone, 4 weeks after the last immunization. Results are shown as the individual values of 4 mice per group, with average and SD. One-way ANOVA with Bonferroni’s multiple comparisons test was used to determine statistical significance among groups, indicated by horizontal bars; ***p<0.001, ****p<0.0001. (TIF) [file pone.0247990.s001.tif]

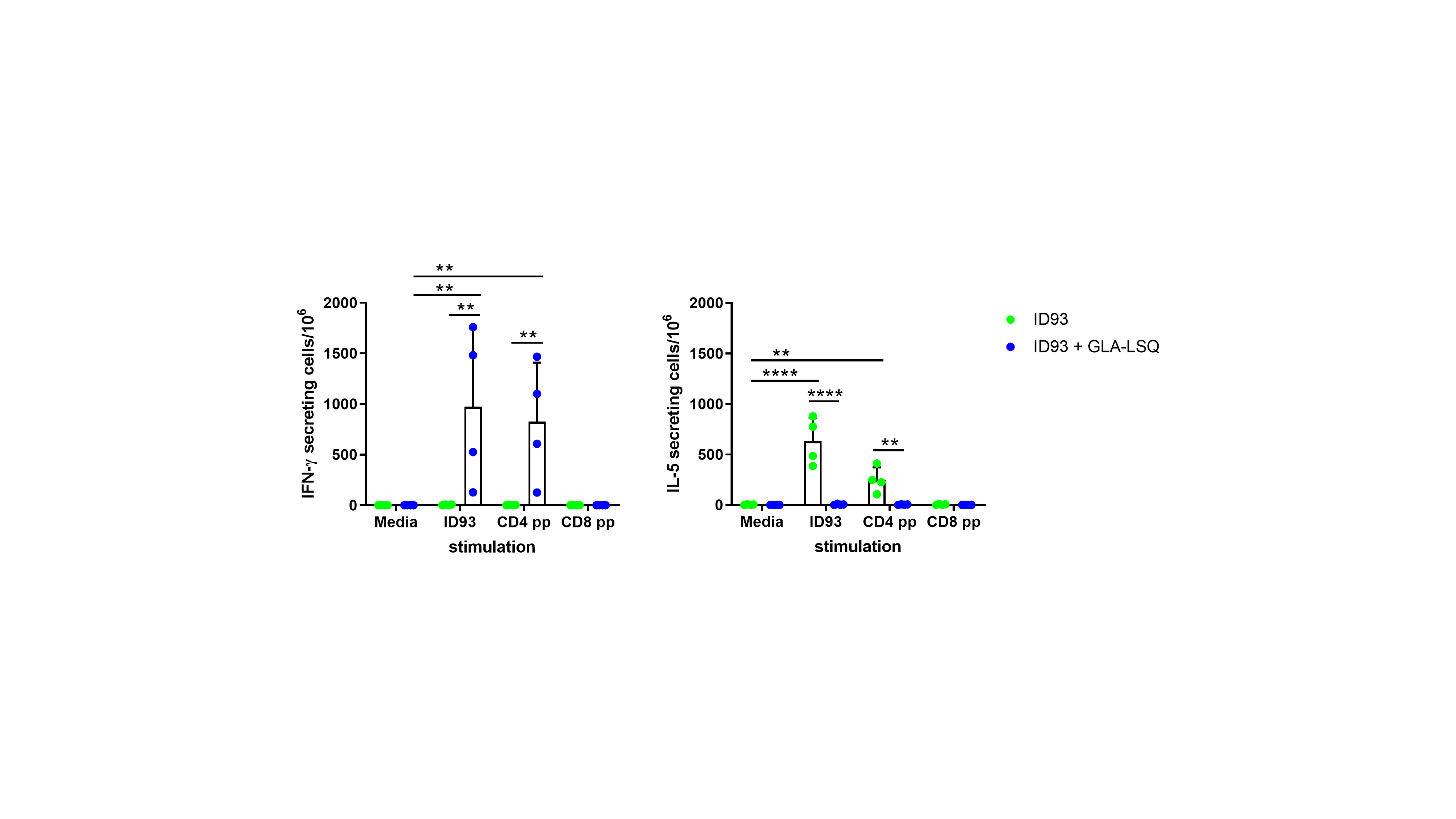

Supplement: S2 Fig — C57BL/6 mice were immunized three times, three weeks apart with ID93 or ID93+GLA-LSQ. Four weeks after the last immunization, spleens were harvested from 4 mice per group, and stimulated with ID93 (10 μg/mL), an ID93 CD4 peptide pool (1 μg/mL) or CD8 peptide pool (1 μg/mL). An (A) IFNγ or (B) IL-5 ELISPOT was performed as previously described [25]. Comparisons were performed using a 2-way ANOVA of ID93 versus ID93+GLA-LSQ for each stimulation, and medium versus stimulations for each immunization, with Bonferroni’s multiple comparison test, **p < 0.01, ****p < 0.0001. (TIF) [file pone.0247990.s002.tif]
